# Duplex Renal Collecting System Lithiasis: A Case of a Challenging Management Technique

**DOI:** 10.1155/criu/1543018

**Published:** 2025-06-12

**Authors:** Ioannis Loufopoulos, Clarence Zwengunde, Soumendra Datta

**Affiliations:** Urology Department, Colchester General Hospital, East Suffolk and North Essex NHS Foundation Trust, Colchester, UK

## Abstract

Duplex renal collecting system is a relatively common congenital abnormality affecting equally both pelvicalyceal systems. Although usually it is an incidental finding, it can cause significant problems to the patients such as recurrent urinary tract infections, hydronephrosis, and lithiasis. In this study, we describe an interesting surgical management option for a patient with upper moiety hydronephrosis and lithiasis of the aberrant ureter, achieving resolution of the hydronephrosis and complete removal of the calculus. A 49-year-old female patient presented with symptoms of left colicky pain. During the initial investigation, a left-sided duplex renal collecting system with severely hydronephrotic upper pole moiety and grossly dilated tortuous ureter with distal calculus and ectopic insertion to urinary bladder was identified. On cystoscopy, the upper moiety ureteric opening was identified distally to the urethral sphincter. Under ultrasound guidance, endoscopic transvesical resection was performed distal to the stented lower moiety ureteric orifice, resulting in the identification of the stone and extraction. Short- and long-term follow-up demonstrated no recurrence of the stone and significant resolution of the hydronephrosis. In conclusion, in this case report, we describe an unusual anatomical variation of the upper moiety outflow, and we introduce a new technique of intravesical ultrasound-guided removal of an obstructive calculus.

## 1. Introduction

A duplex renal collecting system is defined as a kidney unit consisting of two pelvicalyceal systems. Overall, this common congenital abnormality is present in 2%–4% of the adult population, with both left and right kidneys being equally affected. Female patients are reported to be affected twice as likely as males. However, this system can be underreported [1, 2]. Due to the abnormal location when the secondary ureter joins with the bladder, several symptoms may occur when the secondary ureter is ectopic: recurrent urinary tract infections (UTIs), hydroureteronephrosis secondary to nephrolithiasis, and potential urinary incontinence. While duplex collecting systems can cause minor disturbances, upper moiety hydroureteronephrosis and lower moiety vesicoureteral reflux are the most reported findings [3].

The aim of the case report is to retrospectively review the different clinical resolutions of this specific patient with a duplex collecting system and symptomatic relief of abdominal pain due to the obstruction in the aberrant ureter using a unique modality.

## 2. Case Report

**Figure 1:**
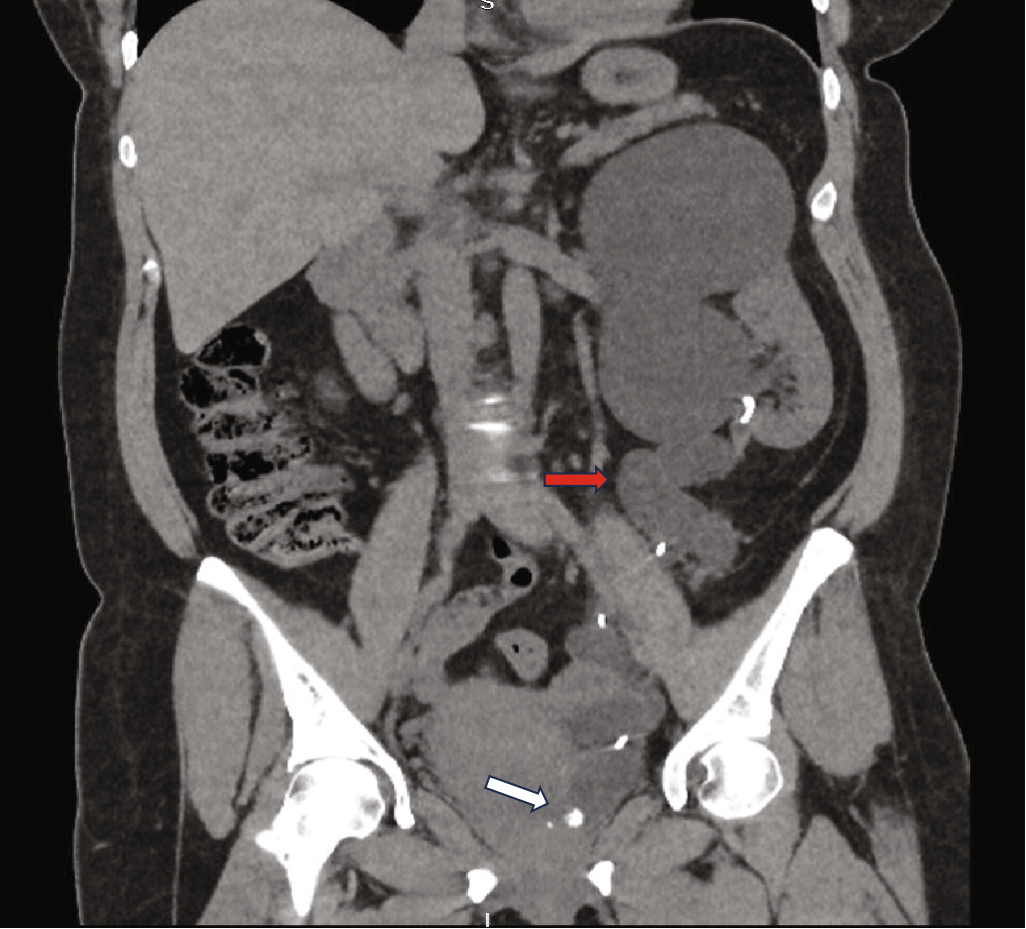
Preoperative CT-KUB: coronal section. Dilated tortuous ureter from upper hydronephrotic moiety (red arrow). Distal calculus on upper moiety dilated ureter (no clear point of upper moiety ureteric orifice on the CT scan—white arrow).

Our patient is a 49-year-old female who presented to the emergency department with acute, left flank pain that radiated to the groin. This pain has happened for numerous years but increased in severity in the last year. In addition to this, the vaginal examination reproduced pain suprapubically on bimanual examination. She reported symptoms synonymous with stress incontinence with diaphoresis but demonstrated no constitutional symptoms of malignancy. The patient's past medical history consisted of rectal prolapse treated with rectopexy, anal atresia, and perianal fistula. Family history is significant for renal cell carcinoma on the maternal side of the patient's family. Otherwise, it is unremarkable for urological specific conditions. In terms of investigation, computed tomography (CT) revealed a left-sided duplex renal collecting system with severely hydronephrotic upper pole moiety and grossly dilated tortuous ureter with distal calculus. No clear point of insertion of the upper moiety to the bladder was observed on the CT scan. The left lower moiety consisted of a mild hydronephrotic ureter (Figure 1). The scan report was inconclusive, suggesting mistakenly the possible presence of a ureterocoele within the urinary bladder. The patient remained afebrile and hemodynamically stable and was managed with a course of teicoplanin and analgesia.

Mercaptoacetyltriglycine (MAG3) renogram demonstrated no reflux, but a chronically obstructed left upper pole with left-sided kidney function 25% and right kidney function 75%.

## 3. Procedure and Management

The case was initially discussed in a multidisciplinary setting to explore the feasibility of endoscopic management as an alternative to heminephrectomy, taking into account the patient's age, renal function, and the suspected anatomical complexity. The decision to proceed with an endoscopic approach was undertaken with the expectation that intraoperative findings might aid in delineating the true anatomical configuration and planning the definitive management approach.

Thus, initially, rigid cystoscopy with retrograde study was performed on the left collecting system, and a ureteric catheter was placed within the left lower moiety before subsequently being successfully stented with a 6/24 JJ stent. During cystoscopic urethral inspection, a small opening (ostium) was identified distal to the urethral sphincter. This was cannulated with a ureteric catheter and emerged from the bladder, suggesting that possibly a false passage was inadvertently created during catheterization, allowing the catheter to traverse the periurethral tissues and enter the bladder directly. The ureteric catheter was promptly removed.

**Figure 2:**
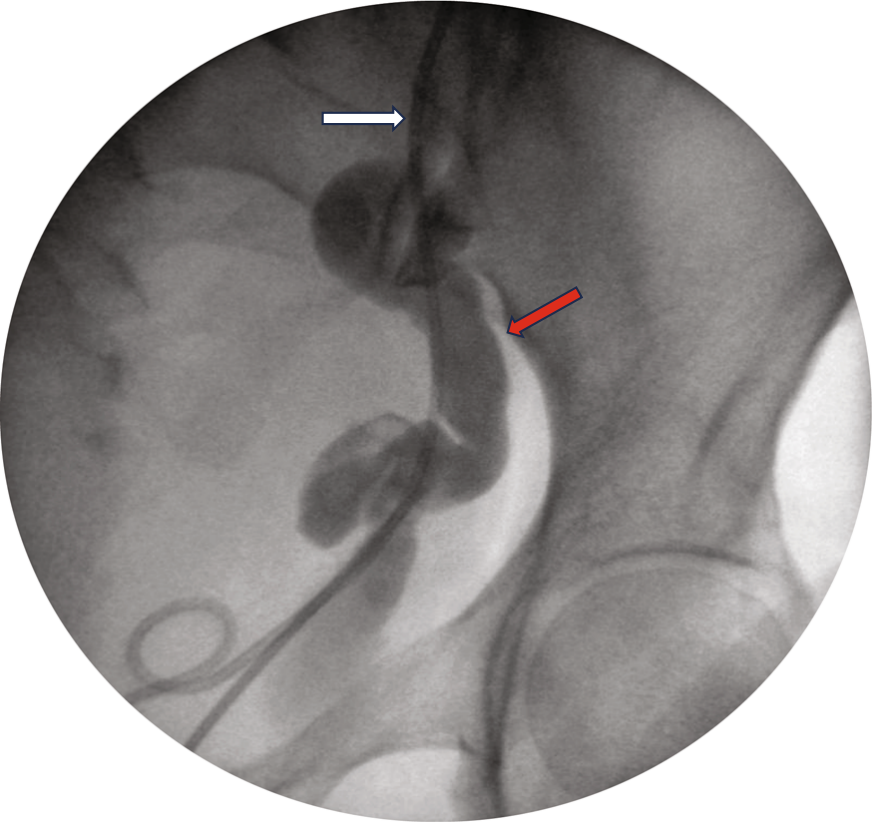
Left retrograde study: dilated left upper moiety ureter (red arrow); left lower moiety ureter stent (white arrow).

Under real-time transabdominal (TA) ultrasound guidance, a transurethral marsupialization of the bladder and the dilated distal upper moiety was performed distally to the stented lower moiety ureteric orifice by resecting a segment of the bladder wall together with the adherent wall of the ectopic, dilated upper moiety. The upper moiety ureter was successfully entered, and it was immediately decompressed with debris-filled fluid emanating from the ureter, indicating chronic obstruction. At the same time, the stone within the massively dilated ureter was revealed and removed with cystoscopic biopsy forceps. Following the creation of the new communication between the bladder and upper moiety, a retrograde study was performed through the newly established opening, delineating the dilated tortuous nature of it (Figure 2). This minimally invasive approach was favored given the significant surgical risks associated with open pelvic surgery in a patient with prior pelvic operations. Recognized risks of the technique included bladder wall perforation with potential injury to vesical arterial branches and the development of a persistent urinoma at the bladder–ureter interface.

**Figure 3:**
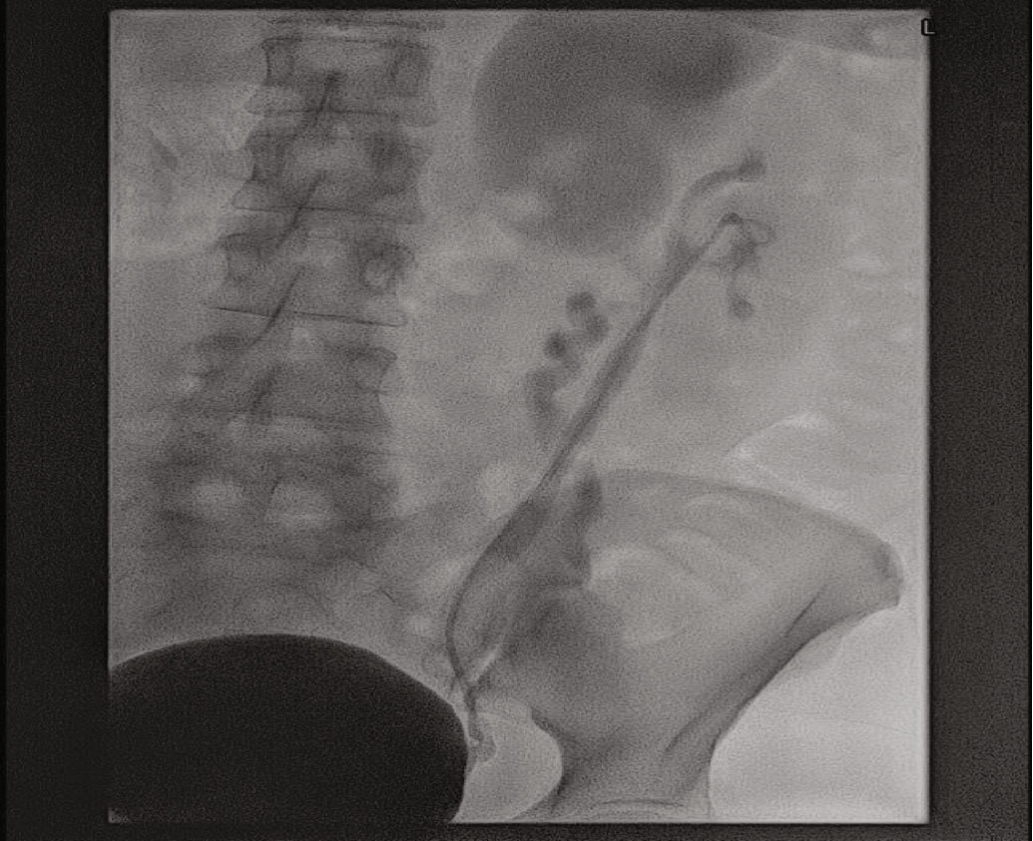
Cystogram performed 10 days postoperatively: no postoperative leakage.

**Figure 4:**
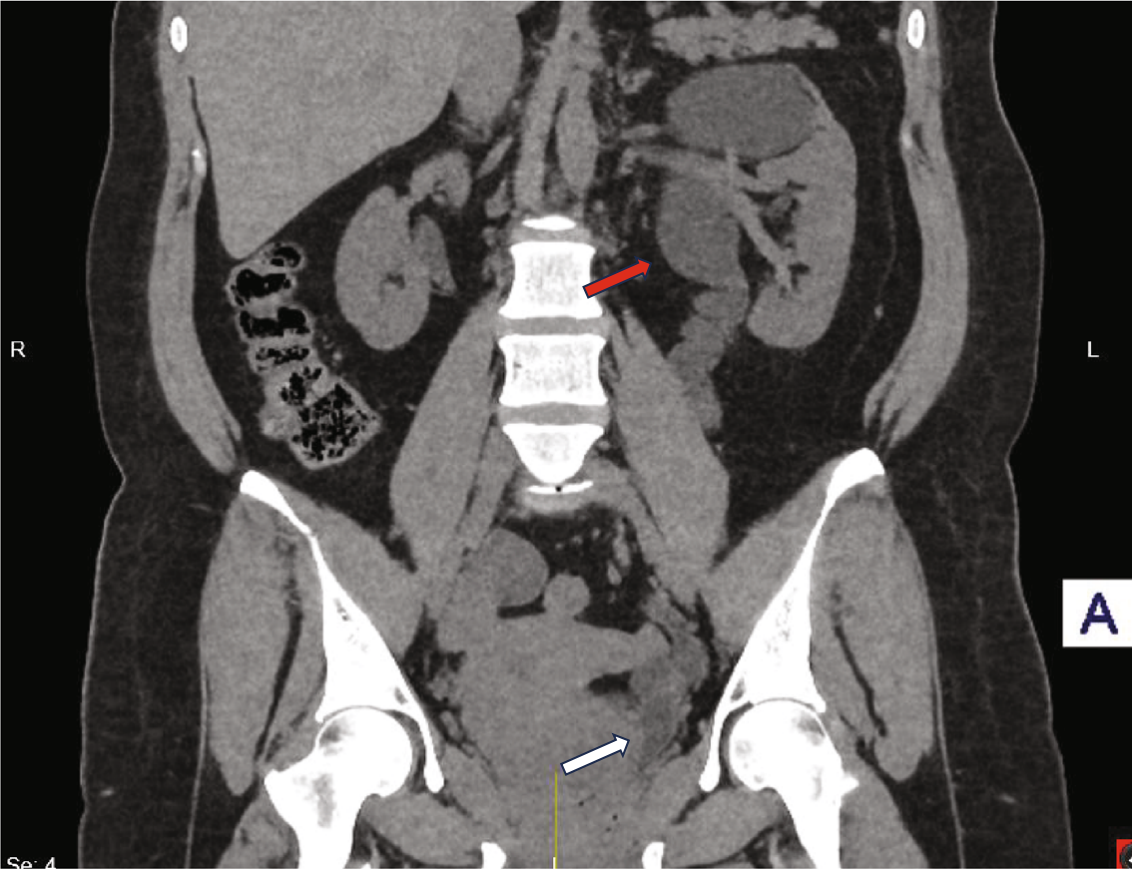
CT urinary tract, 2 months postoperatively: coronal section. Persistent dilated upper moiety (red arrow). No evidence of any remaining calculus (white arrow).

**Figure 5:**
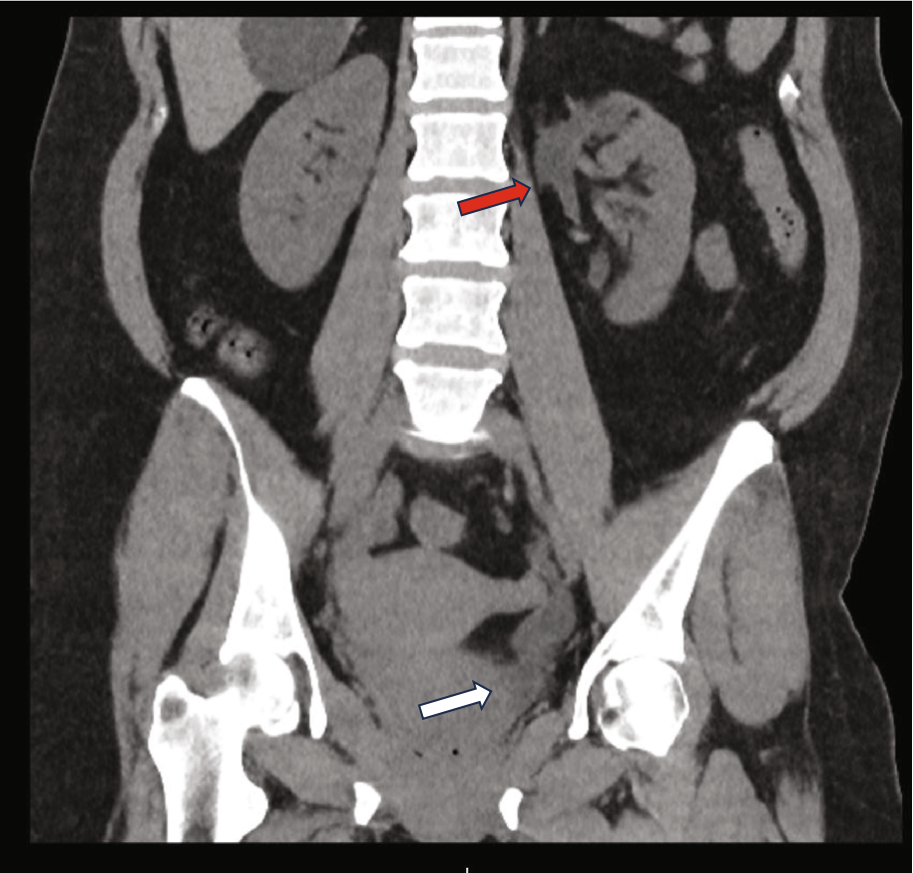
CT urinary tract, 1 year postoperatively: coronal section. Resolving upper moiety dilatation (red arrow). No evidence of the previous calculus (white arrow).

Subsequently, the patient was catheterized for 10 days. There were no intraoperative complications. The urethra catheter was removed 10 days postoperatively following the cystogram, which showed dilated upper moiety and ureter, patent and stented lower moiety ureter, and no leakage from the bladder or the urethra as the ureteric catheter was placed postsphincterically during the operation (Figure 3). The lower moiety stent was removed successfully 3 weeks postoperatively, and the CT urogram 2 months later demonstrated no evidence of calculus but persisting upper moiety dilatation (Figure 4). The patient's symptoms have successfully been resolved 2 months postprocedure. Ultimately, 1 year postoperatively, the patient is free of any remaining stones while the left upper moiety dilatation is significantly resolving (Figure 5).

## 4. Discussion

Renal duplication system is the most common upper urinary tract congenital anomaly and it is usually an incidental finding. Unless there is a complication, the findings of a duplex are incidental. Embryologically, a duplicated collecting system can be one-sided or bilateral and subsequently can be categorized as incomplete or complete. The latter is associated with the existence of an accessory ureteric bud or divided from the solitary one before it reaches its metanephrons [3].

Unless complicated, the majority of them require no surgical intervention [3, 4]. Among the complications, the most common and important ones include recurrent UTIs, vesicoureteric reflux (mainly in the lower moiety), ureteric lithiasis and obstruction, ureterocoele, and subsequently renal impairment and/or urosepsis [5]. As per Fernbach et al., vesicoureteral reflux, ectopic ureterocele, or ectopic ureteral insertion are mostly related to complete duplication, while the latter two affect more frequently the upper moiety [6]. Lithiasis of the duplex system is the commonest one, with an incidence of 4%–20% [7]. In our case, initial CT scans demonstrated an image of ureterocoele at the site of the upper moiety, which eventually was confirmed to be related to ureteric calculus.

Usually, the insertion of dual ureters in the bladder in a completely duplicated system follows the Weigert–Meyer law, according to which the upper moiety ureter (most frequently the ectopic) inserts into the bladder more inferomedially compared to the lower one, which inserts more superolaterally [8]. In our case, the anatomical position of the ureteric orifice imposed the risk of postoperative urinary incontinence as it was distal to the urethral sphincter, which ultimately was avoided as confirmed by the follow-up cystogram. Intraoperatively, the identified ostium was cannulated with an open tip ureteric catheter and subsequently emerged into the bladder. In retrospect, and based on the anatomical findings and clinical context, it is likely that the cannulation of this ostium resulted in the creation of a false passage, allowing the catheter to traverse periurethral tissues and enter the bladder directly. Given the absence of dribbling incontinence over 45 years and the lack of evidence of a natural communication between the dilated ureter and the bladder, a pre-existing patent ectopic orifice was unlikely. Although the catheter advanced smoothly without resistance, suggesting a true lumen at the time, the formation of a false tract remains the most plausible explanation for these intraoperative findings.

In terms of avoiding similar complications, the use of ultrasound was significant, as it facilitated the intraoperative identification of the stone and the exact position of the incision, allowing the upper moiety ureter to be reached uneventfully. We used TA ultrasound to identify the ureter, scope, and bladder. TA is a relatively easy modality of imaging, readily available for use in theatre. TA ultrasound is used in PCNL access routinely. It allows the user to identify the bladder, scope, and the position of the ureters. TA US allowed the surgeon to identify and map the distal part of the upper moiety, which was pivotal, ensuring safe catheterization and deroofing of it without injuring the surrounding structures.

Duplex systems can be complicated with obstruction (more commonly the lower moiety), VUR, ureterocoele, and ectopic ureter. In such cases, personalized treatment is deemed necessary. As described by Keene and Subramaniam [9], reconstructive surgery constitutes the method of choice and can be divided into either upper urinary tract approach (heminephroureterectomy or complete nephroureterectomy) or lower urinary tract approach (bladder reconstruction surgery involving ureterocoele excision and ureteric reimplantation). Although through the latter one, patients demonstrate fewer complications and a lower percentage of reintervention (5% vs. 21%, both approaches are considered major surgeries and particularly challenging for the clinician and the patient). Under this perspective, less invasive techniques are preferred as first-line management options such as transurethral endoscopic incision in cases of infected obstructive systems or ureterocoeles.

In terms of lithiasis of duplex collecting system, it can be managed either cystoscopically, or with ESWL, PCNL, or even total nephrectomy if the kidney function is massively affected. To our knowledge, this is the first case reported, describing the technique of cystoscopic stone removal from a duplex ureteric system with the use of ultrasound and endoscopic puncture and drainage. The skills of percutanous access were used to endoscopically to find the closest point to the bladder allowing drainage of the upper moiety ureter prespincterically into the bladder rather than postsphincterically. This avoids major reconstructive open surgery to divert the ureter.

An iatrogenic injury of the sphincter and subsequent urinary incontinence was considered the main complication of our case and was successfully avoided with complete resolution of the patient's symptoms from the urinary tract stone.

## 5. Conclusion

Duplex renal/collecting systems is a rare urological entity whose complications, and especially lithiasis, can present with atypical clinical symptoms. High suspicion is required by the clinical doctor, as its diagnosis and management can be significantly complex. Our case report describes a rare anatomical variation of the upper moiety outflow and a new technique of intravesical ultrasound-guided drainage as a potential management option.

## Data Availability

The data supporting the findings of this case report are available from the corresponding author upon request.
